# Supplementary material for: Ethnobotany, Phytochemistry, Biological, and Nutritional Properties of Genus Crepis—A Review
Source: Plants (Basel). 2022 Feb 14;11(4):519. doi: 10.3390/plants11040519 (PMC8875603; doi:10.3390/plants11040519)
Supplement: Supplementary file 1 [file plants-11-00519-s001.zip › plants-1592608-supplementary.pdf]

Table S1. *Crepis* taxa studied so far and their synonyms. Accepted botanical name in bold according to The Plant List [2]

***C. alpestris* (Jacq.) Tausch**	*C. grandiflora* Willd.; *C. longifolia* Hegetschw.; *C. mucronata* (Bertol.) Nyman
***C. alpina* L.**	
***C. aspera* L.**	
***C. aurea* (L.) Cass.**	*C. kitaibelii* Froel.
***C. biennis* L.**	
C. bivoniana Soldano & F. Conti	***Crepis vesicaria* subsp. *bivonana* (Soldano & F.Conti) Giardina & Raimondo**; *Crepis purpurea* (Biv.) Steud.
C. bocconi P. D. Sell.	***C. pontana* (L.) Dalla Torre**
***C. bursifolia* L.**	*Crepis balbisiana* (DC.) F.W.Schultz
C. cameroonica Babcock ex Hutchinson & Dalziel	***C. newii* subsp. *oliveriana* (Kuntze) C.Jeffrey & Beentje**
***C. capillaris* (L.) Wallr.**	*C. agrestis* Waldst. & Kit. ex Willd.; *C. bauhiniana* Tausch; *C. candollei* Sch.Bip.; *C. diffusa* DC.; *C. druceana* Murr ex Druce; *C. gaditana* Boiss.; *C. leiosperma* DC.; *C. linifolia* Thuill.; *C. longepinnatifida* Chevall.; *C. lusitanica* Boiss.; *C. neglecta* M. Bieb.; *C. nemorum* Pourr. ex Willk. & Lange; *C. parviflora* Moench; *C. pinnatifida* Willd.; *C. uniflora* Thuill.; *C. variabilis* Krock.; C. *virens* L.
***C. carbonaria* Sch. Bip**	*Crepis ellenbeckii* R.E.Fr.; *Crepis glandulosissima* R.E.Fr.; *Crepis scaposa* R.E.Fr.
***C. commutata* (Spreng.) Greuter**	*C. brachypappa* Bornm.; *C. foetida* f. *bulgarica* (Velen.) Babc.; *C foetida* subsp. *commutata* (Spreng.) Babc.; *C. foetida* f. *gracilis* (Freyn & Sint.) Babc.
***C. conyzifolia* (Gouan) Kern**	*C. amphisboena* Gand.; *C. balcanica* Velen.; *C. djimilensis* K. Koch; *C. grandiflora* Ledeb.; *C. orbelica* Velen.; *C. perrieri* Gand.; *C. sagittata* Wender.; *C. trojanensis* Urum.
***C. crocea* (Lam.) Babcock**	C. czuensis Serg.; *C. gmelinii* var. *grandiflora* Tausch; *C. pallasii* var. *pallasii* Ledeb.; *C. turczaninowii* C.A.Mey.
***C. dioscoridis* L.**	*C. bergeri* (Sch. Bip.) Steud.; *C. croatica* Hornem.; *C. dentata* Gaterau, Descr.; *C. heterosperma* Spreng.; *C. pterosperma* Hort. ex DC. *C. tomentosa* Moench; *C. tubaeformis* Halácsy
***C. divaricata* Boss. & Heldr**	
C. flexuosa (Ledeb.) Benth. ex C.B.Clarke	***Askellia flexuosa* (Ledeb.) W.A.Weber**; *Crepis glauca* Hook.f.; *Crepis kusnezovii* Pavlov; *Crepis melanthera* C.H.An
***C. foetida* L.**	*C. amygdalina* Lag.; *C. eritreensis* Babc.; *C. fallax* Boiss.; *C. foetens* Link; *C. gracilis* Lej.; *C. graveolens* (G.Gaertn. & al.) Schrad. ex Steud.; *C. insularis* Moris & De Not.; *C. interrupta* Sm.; *C. kotschyana* C.B.Clarke; *C. prostrata* (Dumort.) Michot; *C. radiata* Nyman; C. radicata Sm.; *C. rodigioides* Sch.Bip.; *C. schimperi* (Sch.Bip. ex A.Rich.) Schweinf.; *C. thomsonii* Babc.; *C. thracia* Spreng.; *C. zacinthia* (Margot & Reut. ex DC.) Nyman
***C. foetida* L. subsp. *rhoeadifolia* (Bieb.) Celak.**	*C. interrupta* Sieber ex DC.; *C. nemetzii* Rech.f.; *C. nestmeieri* F.Herm. & Degen; *C. rhoeadifolia* M.Bieb.; *C. stribrnyi* Velen.
***C. froelichiana* DC.**	*C. praemorsa* subsp. *corymbosa* (Gaudin) P.D.Sell
C. glauca Torr. and Gray	***Crepis runcinata* subsp. *glauca* (Nutt.) Babc. & Stebbins**; *Crepis chamaephylla* Wooton & Standl
***C. hakkarica* Lamond**	
C. hyemalis (Biv.) Cess., Pass. & Gibelli	***Crepis vesicaria* subsp. *hyemalis* (Biv.) Babc.**; *Crepis reflexa* Guss.; *Crepis taraxacifolia* subsp. *hyemalis* (Biv.) Arcang.
***C. incana* Sm.**	
***C. intermedia* Gray**	*C. acuminata* var. *intermedia* (A.Gray) Jeps.
***C. jacquinii* Tausch subsp. *kerneri* (Rech.f.) Merxm.**	*C. kerneri* Rech.f.
C. japonica (L.) Benth.	***Youngia japonica* (L.) DC.**; *C. fastigiata* (Blume) Sch.Bip.*C. formosana* Hayata; *C. lyrata* (Thunb.) C.B.Clarke; *C. taquetii* (H. Lév. & Vaniot) H. Lév.
***C. lacera* Ten.**	*C. erucifolia* Tausch; *C. latialis* Sebast.
***C. leontodontoides* All.**	*C. aethensis* C. Presl; *C. pauciflora* Poir.; *C. triangula* C. Presl; *C. tuberculata* Sch.Bip. ex Nyman
***C. micrantha* Czerep.**	*C. breviflora* Delile ex Steud.; *C. fuliginosa* Webb & Berthel.; *C. muricata* Sm.; *C. parviflora* Pers.
***Crepis mollis* (Jacq.) Asch.**	*C. croatica* (Waldst. & Kit.) Schloss. & Vuk.; *C. hieracioides* Willd.; *C. hieracioides* Willd.; *C. velenovskyi* Domin
***Crepis multicaulis* Ledeb.**	*C. kochii* Tchich.; *C. stoliczkai* C. B. Clarke
***Crepis napifera* (Franch.) Babcock**	
***C. neglecta* L**	*Crepis cernua* Ten. ex Spreng; *Crepis corymbosa* var. *baetica* Willk.; *Crepis fuliginosa* var. *adscendens* Sm.; *Crepis hyoseroides* Sm.; *Crepis polymorpha* var. *stricta* (Scop.) Wallr.; *Crepis spathulata* Lam.; *Crepis stricta* Scop.
***C. neglecta* L. subsp. *corymbosa* (Ten.) Nyman**	*Crepis corymbosa* Ten.; *Crepis fuliginosa* Sm.
C. nemausensis Gouan	***C. sancta* (L.) Bornm.**
***C. occidentalis* Nutt.**	

***C. paludosa* (L.) Moench**	
***C. pannonica* (Jacq.) K. Koch**	*C. blavii* (Asch.) Stadlm.; *C. glochidiata* (Prokh.) Prokh.; *C. latifolia* Balb. ex Pers.; *C. rigida* Waldst. & Kit.
***C. pulchra* L.**	*C. cichorioides* Gand.; *C. costata* Candargy; *C. cylindrica* Cariot & St.-Lag.; C. deloynei Gand.; *C. hispanica* Pau; *C. lapsanifolia* Rchb.; *C. oxyphylla* Gand.; *C. pulcherrima* Grossh.; *C. valentina* Willk.; *C. vendeana* Gand.; *C. youngiformis* K. Koch
***C. pygmaea* L.**	*C. autaretica* Gand.; *C. royi* Gand.; *C. subglabrescens* Gand.
***C. pyrenaica* (L.) Greuter**	*C. amplexicaulis* Schur; *C. austriaca* Jacq.; *C. blattarifolia* St.-Lag.; *C. foliosa* Dulac
***C. reuteriana* Boiss.**	*Crepis pawlowskii* Strid
***C. rhaetica* Hegetschw.**	*C. heerii* Moritzi; *C. jubata* W.D.J.Koch
C. rhoeadifolia M. Bieb.	***C. foetida* L. subsp. *rhoeadifolia* (Bieb.) Celak.**
***C. rubra* L.**	*C. auriculifolia* Froel.; *C. incarnata* Vis.
***C. rueppellii* Sch. Bip**	*Crepis abyssinica* Sch.Bip; *Crepis adenothrix* Sch.Bip.; *Crepis forskalii* Babc.; *Crepis friesii* Babc.; *Crepis ugandensis* Babc.
***C. sancta* (L.) Bornm.**	C. bifida (Vis.) Muschl.; *C. kochiana* Boiss.; *C. nemausensis* Gouan; *C. roylei* (DC.) F. W. Schultz
***C. senecioides* Delile**	*C. arabica* (Boiss. & Reut.) Boiss.; *C. radicata* var. *nuda* Pamp.;
***C. setosa* Haller f.**	*C. agrestis* M.Bieb.; *C. aspera* Suter; *C. bannatica* Willd.; *C. hamata* Vitman; *C. hastata* Kit.; *C. hispida* Waldst. & Kit.; *C. muricata* Vitman; *C. nova* Winterl
***C. sibirica* L.**	*C. ruprechtii* Boiss.
***C. syriaca* (Bornm.) Babc. & Navashin**	*Crepis alpina* var. *syriaca* Bornm.
***C. tectorum* L.**	*C. arvensis* Jáv.; *C. barckhausioides* Rouy; *C. campestris* Schur; *C. integrifolia* Vest; *C. lanceolata* Kit.; *C. murorum* S.G.Gmel.; *C. segetalis* Roth ex Steud.; *C. tectoria* Dulac; *C. tinctoria* Dulac
***C. terglouensis* (Hacq.) Kern.**	*C. hyoseridifolia* (Pers.) Rchb.
***C. tingitana* Ball**	*C. spathulata* subsp. *boetica* (Lange) Nyman
C. thomsonii Babc.	***C. foetida* L.**
***C. vesicaria* L.**	*C. bicolor* Rchb.; *C. hiemalis* Biv.; *C. macrophylla* Desf.; *C. raphanifolia* Willd.; *C. scariosa* Willd.; *C. taraxacoides* Desf.
***Crepis vesicaria* L. subsp. *haenseleri* (Boiss.) P.D. Sell**	*Crepis vesicaria* subsp. *taraxacifolia* (Thuill.) Thell.
***C. vesicaria* L. subsp. *taraxicifolia* (Thuill) Thell.**	*C. cinerea* Desf.; *C. hackelii* Lange; *C. haenseleri* (Boiss. ex DC.) F. W. Schultz; *C. heterocarpa* Nyman; *C. hyemalis* subsp. *hackelii* (Lange) Nyman; *C. intybacea* Brot.; *C. laciniata* Lowe; *C. numidica* Pomel; *C. polymorpha* Pourr.; *C. praecox* Balb.; *C. recognita* Haller f.; *C. rutilans* Lacaita; *C. scabra* Willd. *C taraxacifolia* Thuill.; *C. taurinensis* Willd., *C. umbellata* Thuill.
C. virens L.	***C. capillaris* (L.) Wallr.**
***C. zacintha* (L.) Loisel.**	
